# Mucosal zipper endoscopic resection offers a novel option for the treatment of gastric submucosal tumors

**DOI:** 10.1055/a-2710-6145

**Published:** 2025-10-07

**Authors:** Zhenkai Luo, Rongwei Ruan, Jiangping Yu, Yuan-Han Zhao, Yuanshun Liu, Yujia Zhai, Shi Wang

**Affiliations:** 1 Department of Endoscopy, Zhejiang Cancer Hospital, Hangzhou Institute of Medicine (HIM), Chinese Academy of Sciences, Hangzhou, China

Endoscopic full-thickness resection (EFTR) has become the mainstream endoscopic resection method for gastric submucosal tumors (SMTs). EFTR enables access to deeper lesions by intentionally creating a full-thickness defect, as opposed to relying on accidental perforation 1 2 . Consequently, closure of the iatrogenic defect remains one of the most critical technical challenges associated with EFTR. Most existing techniques involve full-thickness clamping of the gastric wall, which hinders the spontaneous detachment of metal clips. Additionally, these techniques often require specialized instruments and involve complex procedures, resulting in increased material consumption and costs, which limits their widespread clinical application 3 4 5 .

To overcome these limitations, we developed a novel technique called mucosal zipper endoscopic resection (MZER) ( Fig. 1 , Video 1 ). MZER reduces wound closure time and minimizes the leakage of digestive fluids containing gastric bacteria, thereby reducing the risk of abdominal infection and shortening the healing time. Moreover, the “zipper-like” mucosal closure preserves the mucosal barrier, effectively preventing digestive fluid leakage and reducing the risk of delayed perforation. This is particularly advantageous in cases of leiomyomas, where full-thickness resection is often unnecessary, further emphasizing the benefit of MZER in retaining normal tissue. More importantly, MZER does not require specialized equipment or costly consumables, resulting in lower complication rates and reduced costs, thus significantly lessening the economic burden on patients.

**Fig. 1:**
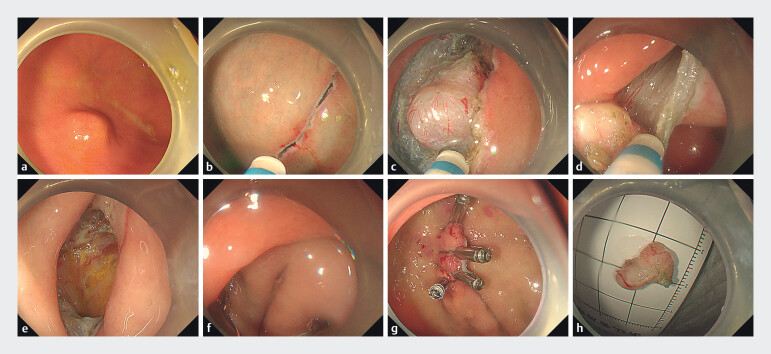
Mucosal zipper endoscopic resection of a gastric submucosal tumor. **a** Endoscopic view of the tumor located in the gastric fundus. **b** Precutting the mucosal layer, forming a longitudinal incision. **c** Separation and full exposure of the tumor. **d** En bloc resection of the tumor. **e** Endoscopic view after tumor removal. **f** Metal clips applied after aligning the mucosal edges. **g** Wound closure with four metal clips. **h** The excised tumor.

Mucosal zipper endoscopic resection performed for a submucosal tumor located in the gastric fundus.Video 1

In conclusion, MZER is a feasible, effective, and safe technique for the treatment of gastric SMTs. Further studies are needed.

Endoscopy_UCTN_Code_TTT_1AO_2AG_3AF

## Notes

Endoscopy E-Videos https://eref.thieme.de/e-videos
*E-Videos* is an open access online section of the journal *Endoscopy* , reporting on interesting cases and new techniques in gastroenterological endoscopy. All papers include a high-quality video and are published with a Creative Commons CC-BY license. Endoscopy E-Videos qualify for HINARI discounts and waivers and eligibility is automatically checked during the submission process. We grant 100% waivers to articles whose corresponding authors are based in Group A countries and 50% waivers to those who are based in Group B countries as classified by Research4Life (see: https://www.research4life.org/access/eligibility/ ). This section has its own submission website at https://mc.manuscriptcentral.com/e-videos .
